# Pragmatic trial assessing polygenic risk driven statin therapy for cardiovascular disease prevention: study protocol for the EE-PRS trial

**DOI:** 10.1136/bmjopen-2026-120048

**Published:** 2026-05-27

**Authors:** Ave Voit, Aet Elken, Margus Viigimaa, Ruth Kalda, Krista Fischer, Karolin Toompere, Alar Irs, Lili Milani, Helene Alavere, Sander Pajusalu, Elin Org, Janika Alloja, Katrin Kaarna, Mikk Jürisson

**Affiliations:** 1 University of Tartu Institute of Family Medicine and Public Health, Tartu, Estonia; 2 North Estonia Medical Centre Cardiology Centre, Tallinn, Estonia; 3 Institute of Clinical Medicine, University of Tartu, Tartu, Estonia; 4 Department of Health Technologies, Tallinn University of Technology, Tallinn, Estonia; 5 University of Tartu Institute of Mathematics and Statistics, Tartu, Estonia; 6 University of Tartu Institute of Genomics, Tartu, Estonia; 7 Heart Clinic, Tartu University Hospital, Tartu, Estonia; 8 Genetics and Personalized Medicine Clinic, Tartu University Hospital, Tartu, Estonia; 9 Clinical Research Center, Tartu University Hospital, Tartu, Estonia

**Keywords:** Primary Prevention, Coronary heart disease, Pragmatic Clinical Trial, Randomized Controlled Trial

## Abstract

**Introduction:**

Atherosclerotic cardiovascular disease (ASCVD) continues to be a leading cause of preventable death globally. Polygenic risk scores (PRS) offer a way to detect individuals at higher relative risk of developing ASCVD, but they have not yet been incorporated into routine clinical practice. Pragmatic trials offer a way to evaluate the integration of PRS into cardiovascular disease prevention in primary care, allowing for a more accurate assessment of their effectiveness in everyday clinical practice.

**Methods and analysis:**

This trial evaluates the effectiveness of preventive statin treatment on reducing the incidence of cardiovascular events and death over 5 years in women (aged 55–80) and men (aged 45–80) with a high coronary artery disease PRS. This is a pragmatic, multicentre, open-label, parallel group, randomised clinical trial with a 1:1 allocation ratio to intervention (n=1350), receiving preventive rosuvastatin 20 mg treatment or control (n=1350), receiving current standard of care. Following the intention-to-treat principle, all randomised participants are analysed according to their allocated group, with the primary outcome defined as time to first major adverse cardiovascular event, comprising ischaemic heart disease, stroke or transient ischaemia, peripheral vascular occlusion and stenosis, revascularisation or cardiovascular death.

**Ethics and dissemination:**

This trial has received ethical approval from the Estonian Committee on Bioethics and Human Research (13.06.2024 nr 1.1-12/1531) and the Estonian Ethics Committee for Medicinal Products (19.12.2024 nr RKU-4/92).

**Trial registration number:**

NCT06820086.

**STRENGTHS AND LIMITATIONS OF THIS STUDY**

- Pragmatic, multicentre, open-label, parallel-group randomised design with 5-year follow-up and primary endpoint ascertainment through national health registries.
- Embedded recruitment within the Estonian Biobank cohort enables nested pharmacogenetic and microbiome sub-studies using pre-existing genetic data and biospecimens.
- Contamination risk due to disclosure of high coronary artery disease polygenic risk scores (PRS) to control arm participants at enrolment, which may prompt independent risk-seeking behaviour despite physician blinding.
- The generalisability of findings to younger high-PRS individuals may be limited given the necessarily older study population required to observe sufficient cardiovascular events.

## Background and rationale

 Atherosclerotic cardiovascular disease (ASCVD) is a significant cause of preventable death worldwide.^1^ Incorporating polygenic risk prediction into the clinical management of ASCVD holds particular importance because of the genetic characteristics of the disease.^2^,^4^

Polygenic risk scores (PRS) are important determinants currently not implemented in the standard clinical risk assessment of cardiovascular disease (CVD).^5^ Studies show that healthy individuals with a very high PRS have a similarly high clinical risk for CVD, as do those with high-impact genetic variants such as those for familial hypercholesterolaemia.^6^,^8^ As genetic testing is not regularly conducted to assess coronary artery disease PRS (CAD PRS), we tend to miss the window of CVD primary prevention among those individuals.^9^

A few focused clinical studies have examined patient responses to CAD PRS communication, finding that genetic risk disclosure alone has not meaningfully changed health behaviour.^10^,^12^ As the atherosclerotic processes develop faster among high-PRS individuals, a more feasible approach could involve combining behavioural interventions with early-onset preventive lipid-lowering treatment with statins.^13 14^

Low-density lipoproteins (LDLs) contribute to ASCVD, and their management with lipid-lowering medications is essential.^15^ Recent studies have found that high PRS amplifies the cardiovascular impact of LDL-C, such that individuals with high PRS and only moderately elevated LDL-C may exceed the treatment threshold for lipid-lowering therapy.^16 17^ Lipid-lowering therapy such as statin treatment has proven to be particularly effective in lowering the LDL of patients with high polygenic risk.^14 18^ Still, the implementation of statin treatment can be challenging as the prescription rate of statins for primary prevention of ASCVD remains low at the primary care level.^19 20^ Significant disparities in controlling LDL cholesterol (LDL-C) levels are evident throughout Europe, with only 20% of patients at high and very high risk reaching the LDL-C goals recommended by the 2019 European Society of Cardiology (ESC).^21 22^ The inadequate prescription of statins is attributed to physicians underestimating the CVD risk in patients and not staying current with the guidelines outlined by the ESC for the prevention and treatment of CVD.^23^

Current research lacks sufficient indication-specific and prospective studies on the utility of PRS for CVD prevention in real-life settings. Our trial seeks to address this knowledge gap by conducting a pragmatic trial where preventive statin therapy is prescribed to individuals with high CAD PRS, aiming to reduce the occurrence of CVD and cardiovascular death.

### Objectives

The primary objective of the EE-PRS trial is to assess the effectiveness of preventive statin treatment based on a high CAD PRS on reducing the incidence of cardiovascular events and death over 5 years in women (aged 55–80) and men (aged 45–80).

Secondary objectives include assessment of all-cause mortality, cardiovascular risk factor modification and treatment adherence and acceptability. The trial also includes pharmacogenetic and microbiome substudies and a cost–utility analysis of PRS-guided statin therapy compared with standard care.

## Methods

### Trial design

This is a pragmatic, multicentre, open-label, parallel group, randomised clinical trial with a 1:1 allocation ratio to intervention (n=1350), receiving preventive rosuvastatin 20 mg treatment or control (n=1350), receiving current standard of care.

### Trial setting

This clinical trial adopts a pragmatic approach as it aims to evaluate the real-world effectiveness of polygenic risk-based preventive statin therapy within Estonia’s primary care system. Conducted over 5 years across approximately 300 primary care practices, the trial leverages Estonia’s unique healthcare infrastructure, which includes a robust primary care network and the Estonian Biobank (EstBB) with genetic data from over 200 000 individuals, from which eligible participants are identified and recruited.

Enrolled participants will receive all trial interventions at their own primary care practice. Intervention arm participants may collect their statin prescription at any pharmacy in Estonia, while control arm participants continue to receive standard primary care without the use of a placebo. Estonia’s small size and centralised healthcare system make it an ideal setting for such a trial, enabling efficient implementation and monitoring.

### Eligibility criteria

This trial intervention is aimed at individuals with high (top 20%) CAD PRS, men aged 45–80 years and women aged 55–80 years. Inclusion and exclusion criteria are listed in box 1.

Box 1 **Inclusion and exclusion criteria.**

**Inclusion criteria**

- Men aged 45–80 on 1 January 2025.
- Women aged 55–80 on 1 January 2025.
- CAD PRS top 20% confirmed by the Estonian Biobank.
- The family physician of the study participant has been contracted to participate in the trial.
- Written informed consent has been provided to participate in the trial.

**Exclusion criteria**

- Diagnosed with ischaemic heart disease (I20–I25), stroke or transient ischaemia (I60–64, I69, G45), peripheral vascular occlusion (I65–66, I67.2, I70, I73.9), diseases of liver (K70–K77), end-stage renal disease (N18.0), mental and behavioural disorders due to psychoactive substance use (F10–F19).
- Currently using statin treatment or has been prescribed statins in the past 3 years.
- Has a confirmed pathogenic variant associated with familial hypercholesterolaemia (APOB, PCSK9, LDLR genes) as verified by the EstBB.
- Is currently participating in other clinical trials.
- Comorbid physical or mental illnesses that prevent the individual from granting consent or participating in the trial (according to the judgement of the investigator).
- Individuals taking:
  - a combination of sofosbuvir/velpatasvir/voxilaprevir (used to treat hepatitis C);
  - ciclosporin;
  - fusidic acid orally or by injection.
- Individuals with hypersensitivity to the active substance (rosuvastatin or atorvastatin) or its excipients.

### Intervention and comparator

The comparison between the statin treatment arm (intervention) and control group (comparator) is conducted pragmatically within the primary care setting, with no placebo used in the control arm. This design ensures that control participants continue to receive usual care from their family physician while intervention arm participants receive preventive statin therapy on top of standard care. This approach reflects real-world clinical practice and allows for a direct assessment of the added value of integrating PRS-guided statin therapy into routine CVD prevention.

### Intervention arm

The first study visit begins with the family physician explaining the nature of polygenic risk and responding to the participant’s questions. The physician will then proceed to counselling the patient in relation to improving their health behaviours utilising techniques such as motivational interviewing. During the visit, the physician will take baseline measurements and blood analyses of the participant and prescribe statin treatment (rosuvastatin 20 mg, 1 tablet per day), explaining treatment schemes and potential side effects. Intervention arm participants attend next primary care visits at months 3, 12 and 60, at which physical measurements, blood tests and side effect assessments are performed by the family physician.

Between scheduled primary care visits, study nurses conduct telemedicine visits at months 6, 24, 36 and 48 to support participant retention throughout the trial. They are also responsible for monitoring trial medication adherence by tracking statin procurement from pharmacies via a dedicated database and contacting the physician or participant as needed if discrepancies in prescribing or dispensing are identified.

The trial intervention is a fixed-dose statin strategy (rosuvastatin 20 mg once daily). Treatment is not titrated to LDL-C targets, as the trial evaluates the effect of PRS-guided statin initiation and sustained exposure on cardiovascular events rather than the effect of intensified lipid lowering. Where rosuvastatin 20 mg is not tolerated, the predefined adverse reaction algorithm applies. Family physicians retain clinical discretion to manage non-trial cardiovascular risk factors according to current guidelines.

### Control arm

Family physicians of the control arm participants will not be informed of their patients’ participation in the trial until the last visit at month 60. This helps to ensure that the care provided to the control group participants reflects as much as possible real-life primary care practice.

The control arm baseline measurements and blood samples are taken at partner hospitals and laboratories across Estonia, all blood analyses are done centrally at the Tartu University Hospital medical laboratory. Physicians are blinded to the results of baseline control arm measurements and analyses until the end of the trial. At month 60 of the trial, physicians will be informed about their patients who were in the control arm for scheduling a final study visit. During the final trial visit, the physicians will inform participants of their CAD PRS, provide counselling and take final measurements.

### Criteria for discontinuing or modifying allocated intervention

This trial involves investigative medicinal products (IMP) licensed in the European Union and are being used within their licensed range of indications, dosage and form. Therefore, no higher risk than the risk of standard medical care is associated with the IMPs and the EE-PRS trial does not include any formal safety endpoints nor the documentation of adverse events. Therefore, the investigators of this trial will collect and report adverse drug reactions (ADRs), serious adverse reactions (SARs) and serious adverse events (SAEs).

The start of the safety reporting window for the trial is the first prescription of statin treatment and the end point is the end of each participant’s participation in the trial. The safety follow-up will continue until the event is resolved.

The reporting of the ADRs can be based on participant reported symptoms (muscle symptoms or oedema) or based on changes to laboratory parameters (creatine kinase, serum-alanine aminotransferase, glycohaemoglobin).

Family physicians follow a predefined algorithm for managing adverse reactions, based on participant-reported symptoms and laboratory findings. Trial medication may be temporarily discontinued, with the subsequent decision to resume rosuvastatin 20 mg or switch to atorvastatin 40 mg once daily depending on whether the adverse reaction resolves.

Family physicians must report the ADR, SAR, SAE on REDCap. The sponsor will then assess the SARs and SAEs and if some are considered Suspected Unexpected Serious Adverse Reactions (SUSARs), report the SUSARs to Eudravigilance following set deadlines.

### Adherence to intervention

As this is a pragmatic trial, we will not be counting issued packages of the study medication or pills taken by study participants in the intervention arm but rather study nurses will be monitoring compliance using a specially designed database on the numbers of issued prescriptions and procured medicines by study participants. The study nurses’ telemedicine appointments will help to ensure better adherence and motivation to take the IMPs by study participants. In addition, qualitative measures of treatment adherence will be used such as the 5-item Medication Adherence Report Scale (MARS-5) in participant online surveys. Statin procurement is also tracked in the real-life control arm via the national prescription database, enabling quantification of contamination during the study period.

### Outcomes

An overview of study objectives and outcome measures has been provided in Table 1.

**Table 1:** Primary and secondary objectives and outcome measures

Objectives	Outcome measures
Primary objective:	Primary outcomes:
To assess the effectiveness of preventive statin treatment based on high CAD PRS on reducing the incidence of cardiovascular events and death.	Time to the first occurrence of Major Adverse Cardiovascular Events, using International Classification of Diseases (ICD-10) codes: ischaemic heart disease (I20–I25), stroke or transient ischaemia (I60–64, I69, G45), peripheral vascular occlusion and stenosis (I65–66, I67.2, I70, I73.9), revascularisation (Z95.1, Z95.5, Z95.8, Z95.9) or cardiovascular death (I00-I78).
Secondary objectives:	Secondary outcomes:
To assess all-cause mortality during the trial period.	Incidence rate of death from any cause among the study participants.
To assess the effect of the trial interventions on lowering cardiovascular risk factors.	Difference in CVD risk factors (LDL-cholesterol, blood pressure, BMI, waist circumference, smoking, alcohol consumption prevalence) by the end of the trial comparing the intervention and control arm.
To assess the adherence and acceptability to preventive statin treatment among high CAD PRS individuals.	Treatment adherence in the intervention arm based on prescriptions and purchases of statins (C10AA, C10BA) and self-reporting using the Medication Adherence Report Scale (MARS-5).^38^ Fidelity of implementation assessed by participant feedback, and adherence to programme activities (record analysis). Acceptability of the primary prevention programme across study participants. Satisfaction with study processes and results.
To assess the plasma concentration and side effects of rosuvastatin therapy depending on the presence of various genetic variants (in SLCO1B1, ABCG2, CYP2C9, CYP2C19, uridine diphosphate glycosyltransferase (UGT) family, SLCO1B3, SLCO2B1, ABCC2, ABCB11 genes).	Difference in plasma concentrations of rosuvastatin comparing study participants with specific genetic variants in the SLCO1B1, ABCG2, CYP2C9, CYP2C19, UGTs, SLCO1B3, SLCO2B1, ABCC2, ABCB11 genes Difference in rosuvastatin side effects between study participants with specific genetic variants in the SLCO1B1, ABCG2, CYP2C9, CYP2C19, UGTs, SLCO1B3, SLCO2B1, ABCC2, ABCB11 genes. Number of participants with adverse events and serious adverse events from statin therapy.
To assess the resource use, cost and cost–utility of preventive statin treatment based on CAD PRS compared with the standard of practice in primary care.	Utilisation of healthcare resources (non-trial physician and cardiologist visits, hospitalisations, length of stay, healthcare and informal resource use, direct healthcare costs). Productivity costs from the iMTA Productivity Cost Questionnaire (iPCQ) .^39^ Number of participants who withdrew or dropped out from the study. Utilities from the EuroQol 5-dimension 5-level questionnaires (EQ-5L-5D) for calculating quality-adjusted life-years. Incremental cost-effectiveness ratio.
To assess the bidirectional relationship between statin therapy and the gut microbiome, specifically focusing on two intertwined aspects: (1) the mechanisms by which statins modulate gut microbiome composition and function and (2) the influence of the gut microbiome on the therapeutic efficacy of statins.	Association between gut microbiome composition and functionality and statin side effect occurrence. Change from baseline in gut microbiome composition and functionality in statin vs control arm. Association between baseline gut microbiome composition and functionality and statin treatment efficacy.

BMI, body mass index; CAD, coronary artery disease; CVD, cardiovascular disease; LDL, low-density lipoprotein; MACE, major adverse cardiovascular event; PRS, polygenic risk score; SAE, serious adverse event.

### Participant timeline

**Figure 1:**
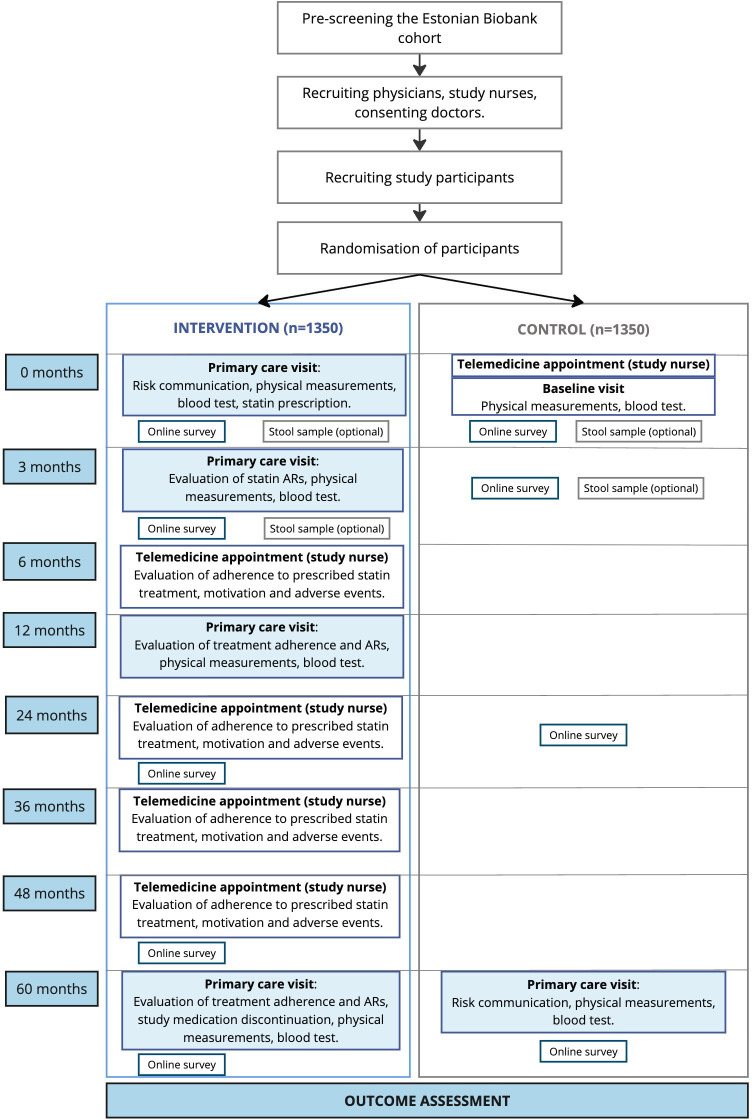
Participant timeline.

The trial runs from March 2025 to March 2031, with each participant followed for 5 years from their enrolment date. A follow-up registry-based analysis will be conducted 3 years after the end of the trial (Figure 1).

### Sample size and power calculation

The power calculations are based on the EstBB cohort, accounting for the observed age and risk factor distribution and the estimated effect sizes. The sampling frame is based on all EstBB gene donors (211 739 individuals).^24 25^ The CAD PRS have been previously calculated for all participants based on the method described by Patel *et al*.^26^

The study target sample includes men aged 45–80 years and women aged 55–80 years who are within the top 20% CAD PRS. The sample used for modelling (n=36 405, 17 861 men, 18 544 women) satisfied the following criteria:

- No prevalent CVD (as defined in the protocol), no missing values in body mass index, systolic blood pressure, smoking level and PRS;
- Age 45–80 at agreement for men and 55–80 for women.

The actual EstBB cohort was used for sample size calculation, assuming that the study sample will be a random sample from all eligible participants. The potential outcome distributions were obtained based on statistical simulation, assuming that the age-specific 5-year major adverse cardiovascular event (MACE) incidence without statin treatment will be similar to what has been observed in the cohort (the Weibull distribution appeared to provide an appropriate approximation) and that the statin treatment will reduce the outcome hazard up to 30%.

Based on an anticipated hazard reduction of 30% with statin treatment and allowing for up to 10% dropout, a recruitment target of 2700 participants is set to ensure a minimum of 80% statistical power.

### Recruitment

The identification of study participants begins with a prescreening of EstBB cohort participants based on set eligibility criteria. As a next step, the primary care physicians with the highest number of potentially eligible patients are contacted to assess their interest in facilitating the trial activities. Primary care centres that agree to participate are contracted for the trial, after which outreach to potentially eligible individuals will begin.

The EstBB will send out invitation emails and SMS messages to potentially eligible individuals, including a link to the MyGenome portal, where they can read recruitment materials describing the trial and its interventions or watch short videos where research team members present the study in an easy-to-understand language and format.^27^

On reading about the study, the individuals must indicate by ticking a box in the MyGenome portal whether they would like to be contacted by a study nurse. All individuals who show interest in participation are contacted via phone call by a study nurse who screens for eligibility and if the individual passes, books them an appointment for a final eligibility assessment and consenting meeting with a study physician. These meetings take place either as a video conference call or in person during which the physician taking the consent will ask eligibility questions from the potential participant and note them down in the study portal. Only individuals who pass this final face-to-face eligibility screening with the physician are then eligible to provide informed consent (either by signing the informed consent form (ICF) on paper or digitally in the MyGenome portal).

The ICF includes two optional questions: one asking participants whether they wish to provide stool samples at months 0 and 3 for microbiome analysis, with samples stored indefinitely at the EstBB for future research; and one asking whether they consent to their registry data being used in a 3-year post-trial follow-up analysis. Only data from participants who explicitly opt in will be included in the follow-up analysis.

Once an eligible participant has signed the ICF, they will be automatically randomised to the intervention or control arm and their eligibility data will be sent from the EstBB database to the trial database REDCap.

### Randomisation

The randomisation unit is an individual participant. The statistician will use a computer-generated randomisation algorithm (permuted block technique) to generate the randomisation sequence. The allocation sequence will be generated by a statistician independent of participant eligibility assessment and enrolment.

The EstBB then uses the randomisation sequence to automate participant allocation to study arms. On signing the ICF, each eligible participant is automatically assigned to either the intervention or control arm and their eligibility data transferred from the EstBB to the trial database REDCap.

### Blinding

Blinding will not be feasible in this trial, but the physicians of participants randomised to the control group will not be communicated about their patients’ participation in the trial. This is necessary to limit the potential influence on the physician to start statin therapy in control subjects. It is still possible that some control group participants start actively seeking statin therapy from their physician or elsewhere. However, we assume that the proportion of these active individuals will not be high and will not modify the overall study outcomes.

### Data collection methods

All study participant data are collected and stored in the REDCap database, with a dedicated data collection form completed for each participant at every study visit.^28^ Data stored in REDCap includes information from study visits, telemedicine appointments, safety reporting, participant online surveys.

An overview of data collection methods for both study arms is provided in online supplemental table S1.

### Data management

All trial-relevant documentation is stored electronically in an electronic Trial Master File.

Personal data will remain confidential for the duration of the study as pseudonymisation will be performed by allocating number codes to each trial participant in the study database. The pseudonymisation key will be stored on the EstBB’s restricted access server, which is not connected to the internet. The data stored in REDCap and the code key will be destroyed after 25 years after the end of the study. The health data collected during the study will be added to the EstBB database and stored there indefinitely in accordance with §18 of the Human Genome Research Act.

### Statistical methods

The primary analysis includes all randomised participants, regardless of intervention compliance, according to the group to which they were assigned from the time of randomisation until the occurrence of the primary composite outcome (time to MACE), based on the treatment policy estimand strategy (intention-to-treat principle). The outcomes for individuals reaching the end of the study without an observed MACE or dying due to another cause will be censored in the primary analysis.

All statistical tests will be two-sided, the level of statistical significance (α) will be 5%, and 95% CIs will be used. Statistical analyses of secondary endpoints will not be formally adjusted for multiplicity. Therefore, these results will be interpreted as exploratory. The statistical analysis will be described a priori in a statistical analysis plan.

Each study subject will be followed for a minimum of 5 years. The primary outcome will be evaluated through a registry-based analysis in both the study and control arm. The trial’s endpoint adjudication committee will assess and confirm the validity of all events.

Crude incidence rates per 1000 person-years will be reported as the number of subjects with events over 5 years divided by the total time at risk for the intervention group of interest (study arm/control arm). For subjects with events, the time at risk will be the time of the first event. For subjects without events, it will be the end of the trial, the time of death (without experiencing the event), or withdrawal of the consent.

The intervention effect is measured by the adjusted HR calculated with 95% CIs, using the Cox proportional hazards model adjusted for gender and age. As 5 years is not a long follow-up period, we might not be able to detect departures from proportionality. However, in case the departures are still detected, stratified log-rank tests will be used instead.

Interim analyses will not be conducted by the research team. An independent Data Monitoring Committee (DMC) will periodically review accumulating efficacy and safety data.

After the clinical trial concludes, a 3-year additional follow-up period will start. At the end of this period, an analysis of registry data concerning the primary endpoint (MACE outcomes) will be conducted. Details about the secondary outcome analyses, subgroup analyses, cost–utility analysis and substudies (microbiome and pharmacogenetics) are provided in the full study protocol.

### Clinical endpoint adjudication committee

A Clinical Endpoint Adjudication Committee (CEAC) has been established given the composite nature of the primary endpoint, which requires independent expert review to confirm the validity of all qualifying events. The CEAC comprises three independent cardiologists who conduct annual assessments, blinded to treatment allocation, of primary endpoints identified through registry queries and electronic health records (EHR), adjudicating each event against predefined trial endpoint criteria. CEAC members are bound by strict confidentiality obligations and are prohibited from disclosing any trial-related information to parties outside the committee, including the principal investigator.

### Data monitoring committee

The DMC is an independent multidisciplinary team comprising a biostatistician, a cardiologist and a primary care specialist. The DMC monitors participant safety and data integrity throughout the trial. The operational details of DMC activities will be described in the DMC Charter prior to the start of DMC work.

### Trial monitoring

Study monitoring independent from study team will be performed according to International Council for Harmonisation Good Clinical Practice guidance version E6(R3) by the Clinical Research Centre of the Tartu University Hospital. Monitoring will be performed according to the approved monitoring plan. Mainly central monitoring will be used, if needed, site visits can be performed. Central monitoring includes the monthly monitoring of protocol deviations based on REDCap reports. By the end of each month, a summary of the monitoring results will be provided to the study coordinator. Any identified discrepancies must be resolved within 1 month and are monitored until full resolution.

###  Ethical approval

The EE-PRS trial follows the principles of the Declaration of Helsinki.^29^ This trial has received ethical approval from the Estonian Committee on Bioethics and Human Research (13.06.2024 nr 1.1-12/1531) and the Estonian Ethics Committee for Medicinal Products (19.12.2024 nr RKU-4/92).

All protocol amendments will be communicated to the relevant ethical bodies and the regulatory authority (the Estonian State Agency of Medicines).

### Study governance

This trial is an investigator-initiated (Institute of Family Medicine and Public Health, University of Tartu), academically sponsored clinical study. The EE-PRS trial organisation includes: (1) study coordinating centres (Tartu University Hospital, North Estonia Medical Center), (2) satellite centres (primary care centres across Estonia), (3) drug dispensaries (all pharmacies in Estonia), (4) medical laboratories (Tartu University Hospital medical laboratory, regional hospitals and medical laboratories across Estonia), (5) a data quality monitor, (6) a DMC and (7) a CEAC.

### Patient and public involvement

Patients and the public were not involved in the design, conduct, reporting or dissemination plans of this research. However, a public engagement campaign was conducted to raise awareness and support participant recruitment.

## Discussion

The EE-PRS trial is one of the first large-scale randomised controlled studies to evaluate the impact of polygenic risk-based primary prevention for CVD in a primary care setting among individuals with high CAD PRS.

A key strength of this trial is its pragmatic design, which avoids using a placebo in the control arm, ensuring that all participants continue to receive standard primary care.^30^ This real-world approach allows for a direct comparison between high polygenic risk-guided statin therapy and usual care, offering valuable insights into the feasibility and effectiveness of integrating genetic risk assessment into routine CVD prevention. By embedding the study within the Estonian National Health Information System, we can leverage routinely collected EHRs and administrative data to enhance the comprehensiveness of our analyses.^31^ Exploratory analyses on the pharmacogenetics of rosuvastatin and the effects of statin treatment on the microbiome enhance the value of this trial, facilitated by the availability of extensive data and resources.

Despite its strengths, this study has potential limitations, including the open-label design, which may introduce potential biases in outcome reporting.^32^ The absence of a placebo control is a deliberate trade-off inherent to the pragmatic design: a placebo-controlled trial would substantially reduce real-world generalisability and increase trial complexity and cost, while not resolving the central question of whether PRS-guided prescribing improves outcomes in routine care. A placebo-controlled design would also not preserve effective blinding in this trial, as the rapid and substantial reduction in LDL-C produced by statin therapy would diverge from placebo within months of randomisation and be apparent through routine lipid monitoring. Although control-arm physicians are not informed of their patients’ trial participation until the end of the study period, there is a risk of contamination, as all participants are informed about their high PRS during the consenting process. This awareness may influence their health behaviours or lead them to seek risk mitigation strategies independently. To quantify potential contamination, statin prescription and dispensing data from the Estonian national prescription database will be analysed for both arms. The generalisability may be limited, as we include participants from the EstBB cohort, which predominantly consists of more educated individuals with relatively high socioeconomic status.^25^

As this study focuses on clinical outcomes and aims to assess differences in major cardiovascular events over a relatively short period, we must select a middle-aged and older population where cardiovascular events are more likely to occur within the study timeframe. This limits the generalisability of findings to younger individuals, who may stand to benefit most from early PRS awareness through timely lifestyle modification and preventive intervention to reduce lifetime cardiovascular risk.^33^ Although concerns around adherence, drug interactions and quality of life have contributed to statin underuse in individuals aged 75 and older, emerging evidence from real-world studies and large ongoing trials increasingly supports their benefit for primary prevention in this population.^34^,^37^

## Conclusions

The EE-PRS trial represents a unique opportunity to generate real-world evidence on PRS-guided cardiovascular prevention within a national primary care system. Its findings can support the development of clinical and policy guidance on the use of genetic risk stratification to reduce cardiovascular morbidity and mortality in high-risk individuals.

## Supplementary material

10.1136/bmjopen-2026-120048 online supplemental table 1
