# Supplementary material for: Pragmatic trial assessing polygenic risk driven statin therapy for cardiovascular disease prevention: study protocol for the EE-PRS trial
Source: BMJ Open. 2026 May 27;16(5):e120048. doi: 10.1136/bmjopen-2026-120048 (PMC13218102; doi:10.1136/bmjopen-2026-120048)
Supplement: online supplemental table 1 [file bmjopen-16-5-s001.docx]

**Table S1. Baseline assessment and follow-up visits for intervention and control arm participants.**

|  |  | Staff member | Baseline | 3 months | 6 months | 12  months | 24  months | 36  months | 48  months | 60  months |
| --- | --- | --- | --- | --- | --- | --- | --- | --- | --- | --- |
|  | **Timepoint** |  | T1(+/- 2 weeks) | T2(+/- 2 weeks) | T3(+/- 2 weeks) | T4(+/- 2 weeks) | T5(+/- 4 weeks) | T6 (+/- 4 weeks) | T7(+/- 4 weeks) | T8(+/- 4 weeks) |
|  | **INTERVENTIONS** |  |  |  |  |  |  |  |  |  |
| **I**  **N**  **T**  **E**  **R**  **V**  **E**  **N**  **T**  **I**  **O**  **N** | CAD PRS risk communication | Family doctor | X |  |  |  |  |  |  |  |
|  | Health counselling | Family doctor | X | X |  | X |  |  |  | X |
|  | Physical measurements | Family doctor | X [1] |  |  |  |  |  |  | X [1] |
|  | Blood test | Nurse | X [2] | X [3] |  | X [4] |  |  |  | X [4] |
|  | Prescription of IMP | Family doctor | X |  |  |  |  |  |  |  |
|  | Side effect monitoring | Family doctor |  | X | X | X | X | X | X | X |
|  | Online survey | REDCap | X | X |  |  | X |  | X | X |
|  | Telemedicine consultation | Study nurse |  |  | X |  | X | X | X |  |
|  | Stool sample* | Estonian Biobank | X | X |  |  |  |  |  |  |
| **C**  **O**  **N**  **T**  **R**  **O**  **L** | Telemedicine consultation | Study nurse | X |  |  |  |  |  |  |  |
|  | Physical measurements | Nurse | X[1] |  |  |  |  |  |  | X [1] |
|  | Blood test | Nurse | X [5] |  |  |  |  |  |  | X [6] |
|  | Online survey | REDCap | X | X |  |  | X |  |  | X |
|  | CAD PRS risk communication | Family doctor |  |  |  |  |  |  |  | X |
|  | Health counselling | Family doctor |  |  |  |  |  |  |  | X |
|  | Stool sample* | Estonian Biobank | X | X |  |  |  |  |  |  |

**Table legend:**

*Optional.

1. Blood pressure, weight, waist circumference, height.
2. LDL, HDL, total cholesterol, calculated creatinine clearance (eGFR), liver function markers (ALAT, ASAT), creatine kinase, glycohemoglobin.
3. LDL, HDL, total cholesterol, liver function markers (ALAT, ASAT), creatine kinase, rosuvastatin plasma concentration.
4. LDL, HDL, total cholesterol, liver function markers (ALAT, ASAT), creatine kinase, glycohemoglobin.
5. LDL, HDL, total cholesterol) and glycohemoglobin.
6. LDL, HDL, total cholesterol.
