# Supplementary material for: Pragmatic trial assessing polygenic risk driven statin therapy for cardiovascular disease prevention: study protocol for the EE-PRS trial
Source: BMJ Open. 2026 May 27;16(5):e120048. doi: 10.1136/bmjopen-2026-120048 (PMC13218102; doi:10.1136/bmjopen-2026-120048)
Supplement: Supplementary file 2 [file bmjopen-2026-120048.reviewer_comments.pdf]

PEER REVIEW HISTORY

BMJ Open publishes all reviews undertaken for accepted manuscripts. Reviewers are asked to complete a checklist review form and are provided with free text boxes to elaborate on their assessment. These free text comments are reproduced below.

ARTICLE DETAILS

Title (Provisional)

Pragmatic trial assessing polygenic risk driven statin therapy for cardiovascular disease prevention: study protocol for the EE-PRS trial

Authors

Voit, Ave; Elken, Aet; Viigimaa, Margus; Kalda, Ruth; Fischer, Krista; Toompere, Karolin; Irs, Alar; Milani, Lili; Alavere, Helene; Pajusalu, Sander; Org, Elin; Alloja, Janika; Kaarna, Katrin; Jürisson, Mikk

---

VERSION 1 - REVIEW

**Reviewer**	**1**
**Name**	**Townend, Jonathan**
**Affiliation**	**University of Birmingham, Cardiology, Cardio-Renal Group**
**Date**	**28-Apr-2026**
**COI**	**None**

An interesting study on an imprtant topic with standard design, though no placebo

Comments

1. The use of 'ischemic heart disease' as part of the primary end point is problematic as it includes 'stable angina' which is sometimes diagnosed on vague clinical grounds only without supportive evidence. I would suggest removing ICD 120 and limiting the ICD IHD codes to 121-125 so that the events (predominantly MI) are better defined and more likley to be genuine MACE.

2. The 10% drop out rate is optimistic in a long treatment period trial. Many trials have drop outs of 20-30%, I would suggest a sample size that allows for 20%.

3. The authors do not mention those patients who on conventional risk scoring would often be advised to commence statins. In reality, a polygenic score would not be used in isolation but in addition to risk factor assessment. I suggest that the authors consider revising the design so that if the Qrisk or similar score indicates a 10 year risk of MACE patients are offered a statin rather than randomisation on conventional treatment grounds. Those with

lower risks should be randomised. This would render the results much more applicable and powerful and esnure the trial refeclts current clinical management.

4. The lack of placebo is a weakness. While it makes the study cheaper and easier it is less robust and also increases the possibility that control patients will commence statin therapy during the trial period.

5. How will patients who are on the active rosuvastatin treatment but fail to respond well be managed? Does the trial allow uptitration and or additional lipd lowering treatment?

VERSION 1 - AUTHOR RESPONSE

Reviewer: 1
Prof. Jonathan Townend, University of Birmingham

Comments to the Author:

An interesting study on an important topic with standard design, though no placebo.

Comments

1. **The use of 'ischemic heart disease' as part of the primary end point is problematic as it includes 'stable angina' which is sometimes diagnosed on vague clinical grounds only without supportive evidence. I would suggest removing ICD 120 and limiting the ICD IHD codes to 121-125 so that the events (predominantly MI) are better defined and more likely to be genuine MACE.**

Response:
We thank the reviewer for this point and agree that I20 (stable angina) is a softer endpoint than I21–I25, given that it can be diagnosed on clinical grounds without confirmatory investigation. The composite definition was set at protocol design with reference to the size of the Estonian Biobank cohort eligible for this trial, which is necessarily smaller than the pools available to comparable trials drawing from, for example, UK Biobank. A narrower endpoint definition would have required a larger sample than could feasibly be recruited within Estonia. The protocol has been finalised, ethics-approved, and recruitment is now complete, so the endpoint definition cannot be revised at this stage.
We have nonetheless built in several safeguards that directly address the reviewer's underlying concern about event validity. All primary endpoint events are independently adjudicated by the Clinical Endpoint Adjudication Committee, comprising three cardiologists blinded to treatment allocation, who assess each candidate event against predefined criteria using registry data and electronic health records. This adjudication process is designed precisely to ensure that recorded events represent genuine MACE rather than coded diagnoses lacking supportive evidence. Individual MACE components will be reported separately, so the contribution of any I20 events to the composite will be transparent.

2. **The 10% drop out rate is optimistic in a long treatment period trial. Many trials have drop outs of 20-30%, I would suggest a sample size that allows for 20%.**

Response:
We thank the reviewer for this point and agree that 20–30% dropout is observed in many long-duration primary prevention trials. The 10% assumption in our power calculation was informed by features specific to the Estonian setting that we judged would mitigate dropout risk:

recruitment from an established biobank cohort whose participants have already demonstrated long-term engagement with research; embedded follow-up within routine primary care rather than dedicated trial visits; primary endpoint ascertainment via national health registries, which captures events independent of active participant follow-up and is therefore robust to participants disengaging from study procedures; and structured retention support through scheduled study-nurse telemedicine contacts.

Our previous two trials with high CAD PRS individuals in Estonia, although evaluating lower-intensity interventions, demonstrated that this population is willing to engage with PRS-based research over extended follow-up.

For your reference the mentioned studies:

- *Viigimaa M, Jürisson M, Pisarev H, Kalda R, Alavere H, Irs A, Saar A, Fischer K, Läll K, Kruuv-Käo K, Mars N, Widen E, Ripatti S, Metspalu A. Effectiveness and feasibility of cardiovascular disease personalized prevention on high polygenic risk score subjects: a randomized controlled pilot study. Eur Heart J Open. 2022 Dec 15;2(6):oeac079. doi: 10.1093/ehjopen/oeac079. PMID: 36600884; PMCID: PMC9803971.*
- *Põld, A., Toompere, K., Elken, A. et al. Evaluating the impact of communicating high polygenic risk for coronary artery disease on the health behaviour of young overweight adults: a pragmatic randomised controlled trial. Sci Rep 16, 4907 (2026). https://doi.org/10.1038/s41598-026-35027-1*

The strong recruitment yield of 40% in the current EE-PRS trial (2,700 participants enrolled from approximately 6,800 eligible individuals contacted) further supports the motivation of the participating cohort. The protocol has been finalized, ethics-approved and recruitment is complete, so the sample size and recruitment target cannot be revised at this stage.

3. **The authors do not mention those patients who on conventional risk scoring would often be advised to commence statins. In reality, a polygenic score would not be used in isolation but in addition to risk factor assessment. I suggest that the authors consider revising the design so that if the Qrisk or similar score indicates a 10 year risk of MACE patients are offered a statin rather than randomisation on conventional treatment grounds. Those with lower risks should be randomised. This would render the results much more applicable and powerful and ensure the trial reflects current clinical management.**

Response:

We appreciate this suggestion and considered it carefully, but we believe the current randomised design is the appropriate one to answer the trial's research question.

Our reasoning:

- First, the trial is deliberately pragmatic. It is designed to evaluate the effectiveness of PRS-guided statin prescribing as it would actually be implemented in Estonian primary care — that is, layered on top of routine clinical practice rather than replacing or restructuring it. A tiered design that pre-treats all SCORE2-eligible participants would test an idealised pathway in which conventional risk-based prescribing already operates at full uptake. The available evidence shows this is not the case: in the SANTORINI study only 20% of high- and very-high-risk patients reached guideline LDL-C targets, and the DA VINCI study documented similarly poor implementation across Europe (both cited in our protocol). Restructuring the trial around assumed-optimal conventional risk management would test a counterfactual rather than current reality.

- Second, the population is already enriched for participants whose statin treatment status reflects current practice rather than guideline ideals: anyone with established CVD, prior statin use, or a statin prescription within the past three years is excluded. The remaining cohort is, by construction, the group in whom PRS would in practice be added on top of usual care.
- Third, family physicians in the control arm retain full clinical judgement and may prescribe statins through routine SCORE2-based assessment if they identify the patient as high-risk via standard practice. The control arm is therefore 'standard of care' in the realistic sense, not 'no treatment'.

4. **The lack of placebo is a weakness. While it makes the study cheaper and easier it is less robust and also increases the possibility that control patients will commence statin therapy during the trial period.**

Response:
We agree the absence of a placebo is a limitation and have expanded our discussion of this trade-off. We note, however, that even a placebo-controlled design would not preserve effective blinding in this trial: statin therapy produces a substantial and rapid reduction in LDL-C, typically evident within weeks of initiation, whereas LDL-C in placebo-treated participants would remain at baseline. Because LDL-C is monitored as part of routine cardiovascular risk assessment in primary care, divergence between the arms would be apparent to both physicians and participants within months of randomization, effectively functionally unblinding the trial. A placebo would therefore add substantial complexity, cost, and participant burden without delivering the blinding benefit it nominally provides, while compromising the real-world generalizability that is the central rationale of this study. A further consideration is that, under the current pragmatic design, control-arm participants remain eligible to receive statin therapy from their family physician if clinically indicated under routine risk assessment; a placebo-controlled design would deny them access to care they would otherwise receive in real-world practice.

5. **How will patients who are on the active rosuvastatin treatment but fail to respond well be managed? Does the trial allow uptitration and or additional lipid lowering treatment?**

Response:
Thank you for raising this. We have clarified the protocol in section "*Intervention and comparator*", that the EE-PRS trial does not aim to titrate statin therapy to LDL-C targets. The intervention is a fixed-dose strategy (rosuvastatin 20 mg once daily) selected because the trial's primary research question concerns whether PRS-guided initiation of preventive statin therapy reduces cardiovascular events compared with standard primary care — not whether intensified lipid-lowering achieves a particular LDL-C threshold. This reflects the clinical rationale of the trial: many high CAD PRS individuals have only moderately elevated LDL-C and would not meet conventional thresholds for statin initiation, yet their elevated polygenic risk places them at meaningful absolute cardiovascular risk that warrants preventive therapy. It also reflects the pragmatic design and the underlying mechanism of statin benefit, where the relative risk reduction in cardiovascular events is driven primarily by sustained exposure to therapy rather than the magnitude of LDL-C reduction in any individual patient.

Accordingly, the protocol does not specify uptitration or addition of further lipid-lowering therapy in response to inadequate LDL-C lowering. What the protocol does specify is the management of inadequate tolerance: where rosuvastatin 20 mg is not tolerated, family physicians follow a predefined algorithm allowing temporary discontinuation, resumption, or

switch to atorvastatin 40 mg once daily. This algorithm prioritises maintaining the participant on statin therapy, which is the exposure of interest in this trial. The protocol does, however, accommodate changes in clinical context during follow-up: a participant who experiences a cardiovascular event (endpoint) transitions to secondary prevention under their treating physician, including any clinically indicated intensification of lipid-lowering therapy, with all dispensed medications captured via the national prescription database.
